# Selective Chromogenic Recognition of Copper(II) Ion by Thiacalix[4]arene Tetrasulfonate and Mechanism

**DOI:** 10.3390/molecules25030612

**Published:** 2020-01-30

**Authors:** Shufang Zhu, Lilin Lu

**Affiliations:** 1 College of Resource and Environmental Engineering, Wuhan University of Science and Technology, Wuhan 430081, China; zhushufang@wust.edu.cn; 2 State Key Laboratory of Refractories and Metallurgy, Wuhan University of Science and Technology, Wuhan 430081, China; 3 Hubei Province Key Laboratory of Coal Conversion and New Carbon Materials, School of Chemistry and Chemical Engineering, Wuhan University of Science and Technology, Wuhan 430081, China

**Keywords:** thiacalix[4]arene tetrasulfonate, copper (II), chromogenic recognition, mechanism

## Abstract

Detection of biologically important transition metal ions such as copper by using a simple method is desirable and of great importance. In this work, we firstly reported that water-soluble thiacalix[4]arene tetrasulfonate (TCAS) exhibited selective chromogenic recognition towards copper(II) ion over other transition metal ions. Color change from colorless to salmon pink was observed in TCAS solution, weak bathochromic shift was induced in UV absorption spectrum of TCAS upon addition of copper(II) ion, and the absorbance of characteristic absorption band at 312 nm increased linearly with copper(II) ion concentration. The recognition mechanism of TCAS to copper(II) ion was investigated by a comparative study with calix[4]arene tetrasulfonate (CAS) and time-dependent density functional theory(TD-DFT) study, and the absorption bands were assigned based on transition orbital analysis.

## 1. Introduction

Over the past decades, considerable efforts have been devoted to the development of molecular receptors for the recognition of biologically important transition metal ions. Copper, whose abundance ranks third in the human body, plays an important role in diverse fundamental physiological processes, i.e., the biosynthesis of hemoglobin, bone development, and the regulation of nerve functions [1,2]. Excessive or inadequate uptake of copper will cause several health hazards [3,4]; therefore, it is very important and meaningful to develop simple techniques such as chromogenic recognition, to detect copper in the environment and biological systems.

Calixarenes are one of the most extensive molecular scaffolds for the development of chromogenic reagents for selective recognition towards metal ions, such as alkali/alkaline earth ions [5,6,7,8], transition metal ions [9,10,11,12], lanthanide and actinide ions [13,14,15], and heavy metal ions [16,17,18]. Much attention has been paid in recent years to the detection of Cu^2+^ ion due to its considerable health and environmental demands. Qazi et al. synthesized a calix[4]arene derivative that has a highly selective chromogenic response to copper ion [19], and thiacalix[4]arene-based imino receptors carrying azophenol appendage were found to show highly selective chromogenic sensing for Cu^2+^ ions over other metal ions by Kumar et al. [20]. Chawla et al. have synthesized calix[4]arene bearing Schiff base loop at the lower rim, which showed chromogenic responses to Cu^2+^ [21]; furthermore, a novel calix[4]dicyano-diimidazole thin film has been developed by Rouis et al. for optical sensing Cu2+, whose the limit of detection is about 7.0 × 10^−9^ mol·L^−1^ [22]. The inherent defects of calixarene derivatives such as poor water-solubility and complicated synthesis process, however, prevent them from wide usage in biological and environmental fields. Synthesis-facile and water-soluble chromogenic sensors based on calixarene and its derivatives, which can realize the favorable recognition of copper ion, are desirable.

**Figure 1:**
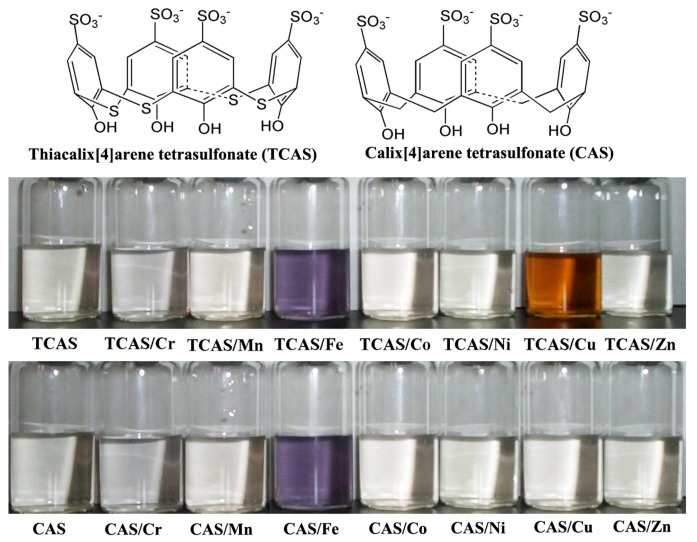
The molecular structures of thiacalix[4]arene tetrasulfonate (TCAS) and calix[4]arene tetrasulfonate (CAS), and color change in aqueous solution of TCAS and CAS upon addition of transition metal ions. The concentrations of TCAS and CAS are both 4.3 × 10^−3^ mol L^−1^, and the concentrations of all transition metal ions are 9.5 × 10^−^^5^ mol·L^−1^.

Thiacalix[4]arene tetrasulfonate (TCAS, Figure 1), a water-soluble derivative of calix[4]arene with a heteratom bridge atom between its phenol units, has been studied in some previous works [23,24]. The crystal structures of a few copper complexes of TCAS have been investigated to clarify the coordination site between TCAS and copper [25,26]. In this work, we firstly reported the highly selective chromogenic recognition behavior of TCAS towards copper(II) ion over other first row transition metal ions. More promisingly, the topic compound TCAS possesses simple preparation procedure and excellent water-solubility. The chromogenic recognition mechanism of TCAS towards copper(II) ion was explored by comparative study with calix[4]arene tetrasulfonate (CAS, Figure 1) and quantum chemical calculations based on time-dependent density functional theory.

## 2. Results and Discussion

### 2.1. Chromogenic Response of TCAS to Transition Metal Ions in Aqueous Solution

The chromogenic response of TCAS to the investigated first-row transition metal ions is displayed in Figure 1. As shown, remarkable color changes were induced by iron(III) and copper(II) ions, TCAS aqueous solution changed from colorless to purple when iron(III) ion was added, and orange appeared after addition of copper(II) ion. However, TCAS showed no distinct chromogenic response towards other transition metal ions, such as Cr(III), Mn(II), Co(II), Ni(II), and Zn(II) ions, demonstrating the selective chromogenic recognition of TCAS towards iron(III) and copper(II) ions. 

It is well known that purple complex can be generally formed between phenol and iron(III) ion; the purple chromogenic response of TCAS was suspected to be the similar response of phenolic compounds to iron(III) ion. To verify this point, control experiments were performed by the addition of iron(III) ion to the aqueous solution of phenol and CAS (Figure 1). As expected, purple presented in both phenol aqueous solution and CAS solution, indicating that the purple chromogenic response is not a distinctive response of TCAS to iron(III) ion. Additionally, copper(II) ion was, respectively, added to phenol and CAS aqueous solutions, but no obvious chromogenic response was observed in them, which indicates that the orange chromogenic response is the distinctive and selective recognition of copper(II) ion by TCAS, which can be easily detected with the naked eye. 

### 2.2. Absorption Spectra of TCAS-Cu Solution and the Effect of Copper Ion Concentration 

**Figure 2:**
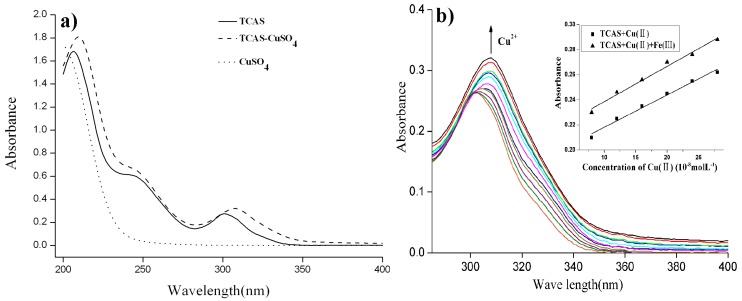
(**a**) Absorption spectra of TCAS (4.5 × 10^−^^5^ mol·L^−1^), CuSO_4_, and TCAS-CuSO_4_ aqueous solution; (**b**) effect of Cu(II) concentration on absorbance at 312nm and linear relation in Cu(II) concentration range of 0.8–2.8 × 10^−7^ mol·L^−1^ with and without Fe(III) ion (shown in the inserts).

UV/Visible absorption spectra of TCAS and TCAS-copper(II) aqueous solution were recorded to investigate the influence of copper(II) ion on the absorption property of TCAS. As shown in Figure 2a, two main absorption bands at 205 and 307 nm appeared in the absorption spectrum of TCAS (4.5 × 10^−5^ mol·L^−1^). In the absorption spectrum of TCAS-copper(II) aqueous solution, the absorption bands shifted to 210 and 312 nm, respectively, meaning approximately 5 nm bathochromic shift of main absorption bands. In addition, distinct enhancement was induced in absorption intensity of the main absorption bands by copper(II) ion.

To explore the origin of the remarkable change in absorption spectra, the UV-vis spectrum of CuSO_4_ aqueous solution was also recorded for comparison. As can be seen from Figure 2a, no detectable absorption feature presented in 250~400 nm in CuSO_4_ absorption spectrum, and the absorption bands were only located in the range of less than 250 nm, which revealed that coordination reaction between TCAS and copper(II) ion, rather than the superimposition effect of the absorption band of TCAS and copper(II) ion, resulted in distinct enhancement of absorption intensity.

The effects of copper(II) ion concentration on absorbance at 210 and 312 nm of Cu(II)-TCAS solution were investigated. Continuous increase in absorbance at 312nm along with the augment of copper(II) ion concentration was observed (Figure 2b), and a linear relations between absorbance and copper(II) ion concentration in the range of 0.8 × 10^−7^ to 2.8 × 10^−7^ mol·L^−1^ was presented. The linear regression equation was determined to be absorbance (A) = 0.002571 × C(10^−8^ mol·L^−1^ Cu(II)) + 0.1924, r = 0.994, n = 6. However, there was no continuous increase in absorbance at 210 nm and no linear relationship between the absorbance at 210 nm and copper(II) ion concentration. To investigate the influence of iron(III) ion, the absorbance of aqueous solution containing 4.5 × 10^−5^ mol·L^−1^ TCAS, 8.0 × 10^−5^ mol·L^−1^ Fe(III) ion, and (0.8~2.8) × 10^−7^ mol·L^−1^ Cu(II) ion, at 312 nm, were measured and shown in the insert in Figure 2b; the result indicates that the presence of Fe(III) ion does not show distinct effect on the linear relations between absorbance and copper(II) ion concentration, despite a slight increase in the absorbance. These results indicated TCAS displayed a highly selective and distinctive chromogenic recognition to copper(II) ion over other transition metal ions, and quantitative detection of copper(II) ion can be implemented in the range of 0.8 × 10^−7^ to 2.8 × 10^−7^ mol·L^−1^ by TCAS.

### 2.3. The Mechanism of Selective Chromogenic Response of TCAS to Copper Ions

By comparison of the molecular structure of TCAS and CAS, possible mechanism of distinctive chromogenic recoginition of TCAS towards copper(II) ion can be inferred as the complexation between TCAS and copper(II) ion via the bridge sulfur atom site. This complexation structure between TCAS and copper(II) ion had been reported in previous experimental work [25,26].

**Figure 3:**
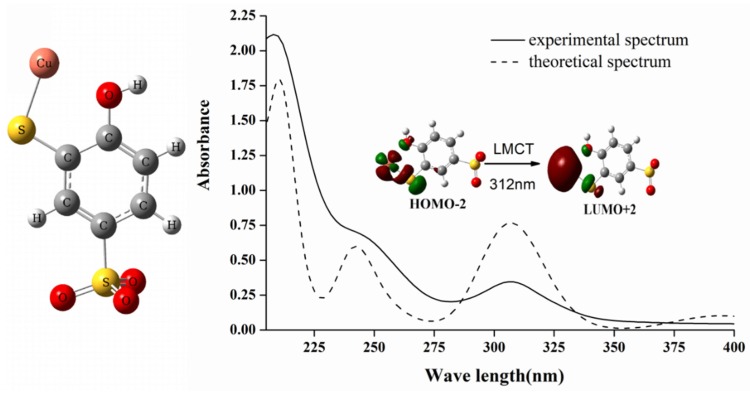
Theoretical model of TCAS-Cu complex (**left**), and experimental and theoretical UV absorption spectrum of TCAS-Cu complex (**right**).

To verify this point, Time-Dependent Density Functional Theory (TDDFT) [27] calculations were performed to investigate the effect of coordination between TCAS and copper(II) ion on the UV/Visible absorption properties. To circulate the vast calculation cost caused by the considerable size of calixarene molecule, a representative computation model of TCAS-Cu complex was selected to perform quantum chemical calculation. The experimental and theoretical absorption spectrum in aqueous solution were displayed in Figure 3, and theoretical absorbance was scaled so as to compare with experimental spectrum.

**Figure 4:**
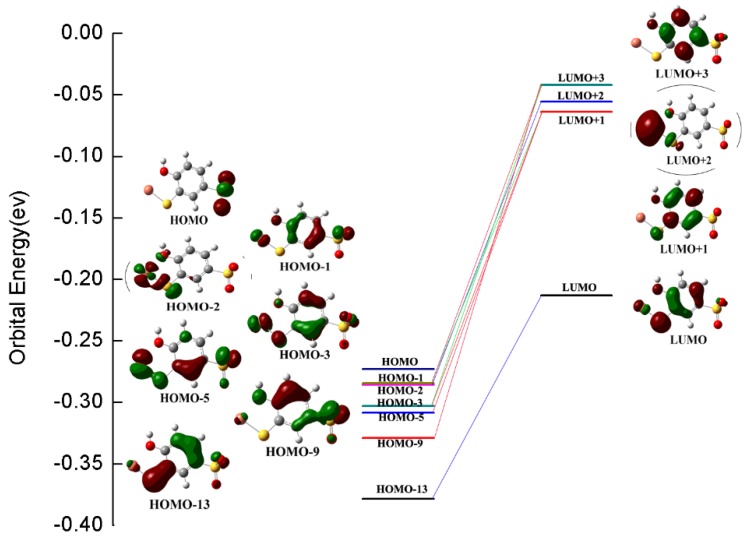
Topologies of molecular orbital involved in electronic transitions of main absorption bands.

As can be seen from Figure 3, the theoretical spectrum was in excellent agreement with experimental spectrum, and the main absorption bands at 210, 242, and 312 nm in experimental spectrum were all accurately reproduced by theoretical calculation. Table 1 lists the three main absorption bands and orbital contributions, and the topologies of selected molecular orbital involved in electronic transitions are shown in Figure 4 to provide deep insight into the nature of UV/Vis absorption bands. The main contributions to 210 nm absorption band were HOMO − 1 to LUMO + 3, HOMO − 3 to LUMO + 1, HOMO − 9 to LUMO + 3, and HOMO − 5 to LUMO + 1 electron transitions; their topological analysis indicated that these orbitals were of π-nature, and that this absorption band can be assigned as π→π* transition of benzene ring. Additionally, metal-ligand charge transfer (MLCT) transition from copper to benzene ring also contributed to this absorption band. The contributions to 242 nm band were HOMO − 1 to LUMO + 1 and HOMO − 3 to LUMO + 3 transitions, and the transition character was similar to the absorption band at 210 nm. The maximum contribution to 312 nm band was HOMO − 2 to LUMO + 2 transition, mainly spread over copper(II) and sulfur. From the orbital topology, HOMO-2 can be described as Cu-S coordination bond orbital; LUMO + 2 was the orbital with copper(II) ion 4s orbital character, and the transition between them can be assigned as ligand to metal charge transfer (LMCT) transition corresponding to 312 nm absorption band.

## 3. Materials and Methods 

Thiacalix[4]arene tetrasulfonate(TCAS) was synthesized according to the methods reported in literature [23,24]. Transitional metal ions Cr(III), Mn(II), Fe(III), Co(II), Ni(II), Cu(II), and Zn(II) were offered in commercial inorganic salt (CrCl_3_, MnSO_4_, FeCl_3_, Co(NO_3_)_2_, NiSO_4_, CuSO_4_, and ZnSO_4_) of analytical grade and used without further purification, and the stock aqueous solutions were prepared by using double distilled water. Five hundred microlitres of stock solution (2.0 × 10^−3^ mol·L^−1^) of transitional metal ions was added to 10 mL TCAS solution (4.5 × 10^−3^ mol·L^−1^) and 10 mL CAS solution (4.5 × 10^−3^ mol·L^−1^), respectively, while constantly stirring at room temperature, and the color changes were recorded immediately. UV/Visible absorption spectra were recorded on a UV-2550 spectrophotometer (Shimadzu, Tokyo, Japan). A certain amount of Cu(II) ion stock solution (2.0 × 10^−3^ mol·L^−1^) was added to 1 mL TCAS solution (4.5 × 10^−3^ mol·L^−1^), and the final volume of the solution was adjusted to 100 mL with double distilled water. Two minutes later, the absorbance was measured at 312 nm in 1 cm quartz cell against water blank at room temperature. 

GAUSSIAN03 program package [28] was chosen to perform geometries optimizations, vibrational analysis, and excitation energy investigation. The stable stationary points of structures were obtained by geometry optimization at B3LYP/6-311+G(d,p) level with the default convergence criteria, and vibrational frequency calculations were performed to confirm all frequency were real at the same computation level. Time-dependent parameter-free PBE0 hybrid functional and 6-311++G(2d,2p) basis sets were chosen to calculate vertical electronic excitation energies without zero-point energy correction, and bulk solvent effect was evaluated by the SCRF method via polarized continuum model (PCM). All solvent parameters are default-implemented in Gaussian program.

## 4. Conclusions

The addition of copper(II) ions to thiacalix[4]arene tetrasulfonate(TCAS) induced a distinct chromogenic response, in which colorless TCAS aqueous solution changed to salmon pink when copper(II) ion solution was added. The main bands in the absorption spectrum exhibited weak bathochromic shift; there was a linear relationship between the absorption band intensity at 312 nm, and copper(II) ion concentration was determined to be absorbance(A) = 0.002571 × C (10^−8^ mol·L^−1^ Cu(II)) + 0.1924 in the concentration range of 0.8 × 10^−7^ to 2.8 × 10^−7^ mol·L^−1^. Time-dependent density functional theory studies revealed that the coordination between bridge sulfur atom of TCAS and copper(II) ion is the origin of the chromogenic response of TCAS to copper(II) ion, and the characteristic absorption band at 312 nm was assigned as ligand to metal charge transfer (LMCT) transition based on transition orbital analysis. 

## Figures and Tables

**Table 1:** Experimental and theoretical absorption bands, oscillator strengths, and orbital transition contributions.

Experimental	Theoretical	
	Absorption Bands	Orbital Contributions
	210 nm (*f* = 0.2851)	
	HOMO − 1→LUMO + 3	0.4233
	HOMO − 3→LUMO +1	0.3141
210 nm	HOMO − 9→LUMO + 3	0.3011
	HOMO − 5→LUMO + 1	0.2744
	242 nm (*f* = 0.0950)	
242 nm	HOMO − 1→LUMO + 1	0.6658
	HOMO − 3→LUMO + 3	0.1144
	312 nm (*f* = 0.0778)	
312 nm	HOMO − 2→LUMO + 2	0.5067
	HOMO − 13→LUMO	0.4104
